# Ancestry-informative markers and variants of uncertain significance on hereditary cancer panels

**DOI:** 10.3389/fonc.2026.1787278

**Published:** 2026-04-29

**Authors:** Maheen Farooqi, Charité N. Ricker, Chenery L. Lowe, Mackenzie D. Postel, Jesus Resendiz, Serina Ovalle, David W. Craig, Bodour Salhia, Julie O. Culver

**Affiliations:** 1 Department of Genetics, Stanford School of Medicine, Stanford, CA, United States; 2 USC Norris Comprehensive Cancer Center, University of Southern California, Los Angeles, CA, United States; 3 Center for Biomedical Ethics, Stanford School of Medicine, Stanford, CA, United States; 4 Department of Integrative Translational Science, City of Hope, Duarte, CA, United States; 5 Department of Cancer Biology, Keck School of Medicine, University of Southern California, Los Angeles, CA, United States

**Keywords:** ancestry, ancestry informative marker, cancer, ethnicity, genetic counseling, genomics, race, variants of uncertain significance

## Abstract

**Introduction:**

Variants of uncertain significance (VUS) on multigene cancer panels can increase patient anxiety and lead to unnecessary interventions. Prior literature shows that, compared to Non-Hispanic Whites, VUS are more frequent among other racial and ethnic groups due to underrepresentation in population genetics databases. However, self-reported race/ethnicity is an imperfect proxy for genetic ancestry. Estimating genetic similarity using ancestry-informative markers may provide a more precise approach to assessing VUS frequency.

**Purpose:**

This study examined the association between genetic similarity and presence of VUS results among patients who underwent hereditary cancer panel testing. A sub-analysis in self-identified Hispanic participants assessed whether Indigenous American similarity was associated with VUS.

**Methods:**

Data were obtained from cancer patients in the Oncology Research Information Exchange Network (ORIEN), a national cancer research alliance. Patients were included if they underwent genetic counseling and multigene cancer panel testing. Genetic similarity was calculated based on ancestry-informative single nucleotide polymorphisms. Each participant received proportion estimates for seven ancestral groups: European, Indigenous American, East Asian, Middle Eastern, African, South Asian, and Oceanian. Predominant genetic similarity was defined as the ancestral group with the highest proportion estimate. Multivariable logistic regression was used to assess the association between genetic similarity and VUS.

**Results:**

The study included 597 participants and 50% had at least one VUS. Self-identified race/ethnicity included: 44% non-Hispanic White, 37% Hispanic White, 14% Asian, 2% non-Hispanic Black, and 3% unknown/multiracial. Self-reported race/ethnicity did not show a consistent association with VUS. Individuals whose predominant genetic similarity was non-European had increased odds of having a VUS compared to those with predominantly European genetic similarity: East Asian (OR = 2.06, 95% CI = 1.20-3.53), Middle Eastern (OR = 1.92, 95% CI = 1.03-3.56), African (OR = 2.48, 95% CI = 0.96-6.42), and Indigenous American (OR = 1.41, 95% CI = 0.92-2.17). Among self-reported Hispanics, when genetic similarity was measured continuously, every 10% increase in Indigenous American similarity was associated with a 11% increase in odds of having a VUS (OR: 1.11, 95% CI = 0.94-1.33).

**Conclusion:**

When assessed using ancestry-informative markers, non-European genetic similarity was associated with significantly increased odds of VUS results. Current reliance on self-reported race/ethnicity may mask the complexity of ancestry-related disparities in VUS occurrence.

## 1 Introduction

Variants of uncertain significance (VUS) are common findings on multigene panels used for hereditary cancer testing. Since 2013, panel sizes have grown, increasing the likelihood of detecting a VUS (1–5). While VUS detected on hereditary cancer panels are typically considered uninformative for clinical management, studies show that they can cause patients significant anxiety and distress, alter patient self-perception, and occasionally lead to unnecessary medical interventions such as risk-reducing surgery or intensified screening, particularly when misunderstood by non-genetics providers (6–11).

It is well established that the burden of VUS findings is not evenly distributed across populations. Individuals with non-European ancestry, including Hispanic, African American, and Asian populations, may be 1.5 to 2 times more likely to receive VUS results on cancer panels compared to individuals of European descent (12–16). Prior literature attributes this disparity to the underrepresentation of diverse populations in reference databases (17, 18). Most studies examining VUS disparities rely on self-reported race and ethnicity (19). However, this approach presents two interrelated limitations. First, the reliance on self-reporting introduces inconsistency, as self-identification can be context-specific, fluid, or missing entirely from clinical records (20, 21). Second, race and ethnicity are social constructs that serve as imperfect proxies for biological ancestry, often failing to capture the genetic complexity of admixed populations (18, 22).

In contrast, genetic similarity, which is estimated using ancestry-informative single-nucleotide polymorphisms (SNPs), offers a biologically-grounded and more objective measure of ancestral background (23, 24). This is particularly relevant for the U.S. Hispanic population, where historical admixture results in varying proportions of European, African, and Indigenous American ancestry that self-identification may not fully capture (25).

Despite these advantages, little research has examined the direct association between genetic similarity and VUS likelihood (19, 26). This study investigates the association between genetic ancestry and VUS burden in a diverse cohort. We determined whether ancestry-informative SNPs are associated with VUS occurrence on a clinical hereditary cancer panel. In a secondary analysis of self-identified Hispanic individuals, we specifically examine whether the proportion of Indigenous American ancestry-informative SNPs influences the VUS rate, allowing us to further assess the burden of VUS among self-identified Hispanic individuals.

## 2 Materials and Methods

### 2.1 Participant selection

Patients were included if they were seen at the University of Southern California (USC) Norris Comprehensive Cancer Center or Los Angeles General Medical Center between 2013 and June 2024, underwent genetic counseling and germline cancer genetic testing, and who had genetic similarity estimates available through the Oncology Research Information Exchange Network (ORIEN), a national collaboration of cancer centers designed to support precision oncology research (27, 28).

Genetic testing was separately performed by certified commercial laboratories. Individuals who received only site-specific or single-gene or single-syndrome testing (e.g., *BRCA1* and *BRCA2* alone) were excluded. All participants had germline test results in one of four categories: negative (benign or likely benign), positive (pathogenic or likely pathogenic variant without VUS), positive with one or more VUS, or VUS only. For this analysis, results were dichotomized into 0 VUS (negative or positive without VUS) versus ≥1 VUS (VUS only or positive with one or more VUS).

Demographic variables such as age, sex and cancer type were obtained from the medical record. All patients reported maternal and paternal ancestry, if known, and a subset of patients filled out a questionnaire in advance of the genetic counseling appointment and self-identified their race/ethnicity. For those who did not fill out a clinic questionnaire, the genetics staff inferred the patient’s race/ethnicity based on the patient’s self-reported maternal and paternal ancestry, using standard US Census categories (29).

### 2.2 Genetic similarity estimation

Genotype data were obtained through ORIEN. ORIEN includes data on cancer treatment, outcomes, tumor genomics, and germline testing, offering linkage of clinical records with tumor and germline genomic results not commonly available in other datasets (27, 28, 30).

Germline DNA underwent whole exome sequencing via Illumina HiSeq4000 or NovaSeq6000 instruments using Roche NimbleGen, IDT, or Twist Bioscience capture kits. Quality control filtering retained biallelic SNPs with minor allele frequencies >= 1% and genotyping rates >= 90% and removed sites with excess heterozygosity. Variant quality score recalibration was applied using *gatk*. Without utilizing imputation, these filtered SNPs were intersected with gnomAD and dbSNP reference sets. This yielded a final panel of 135,472 ancestry-informative SNPs with a 99.4% genotyping rate. Genetic similarity was estimated using an ADMIXTURE-based framework (v1.3.0) trained on 4,150 individuals from the 1000 Genomes Project and Human Genome Diversity Panel. Eight ancestral clusters (K = 8) were specified and supported by 5-fold cross-validation.

This approach generated continuous estimates summing to 1 for reference clusters defined in gnomAD v3.1.2: European, Central African, non-Central African, East Asian, South Asian, Middle Eastern, Oceanian, and Indigenous American. In our cohort, the two African clusters were combined, resulting in seven observed genetic similarity groups across the study population: African, Indigenous American, European, East Asian, Middle Eastern, South Asian, and Oceanian (30). Each participant was categorized into their predominant genetic similarity group, defined as the group with the highest proportional estimate. This approach enables comparison across broadly defined ancestral groups while avoiding the application of arbitrary threshold, for which no consensus currently exists in the literature (31, 32).

### 2.3 Statistical analysis

The primary outcome was defined as the presence of one or more VUS (≥1 VUS vs. no VUS). Covariates expected to confound the relationship between VUS identification and predominant genetic similarity, including the number of genes tested (as testing for more genes increases the possibility of detecting a VUS), year of testing (to account for variant downgrades and upgrades over time), and testing laboratory (reflecting lab-specific classification criteria), were first evaluated in univariate analyses. Covariates significantly associated with VUS were included in the multivariable logistic regression models. One testing laboratory (University of Washington) was omitted as a discrete covariate level due to sparsity (n=1) and was instead evaluated within the reference group.

Two multivariable logistic regression models were performed, with presence of ≥1 VUS as the dependent variable and 1. self-reported race/ethnicity as the independent variable in the first model and 2. predominant genetic similarity as the independent variable in the second model.

Results from the regression analyses were reported as odds ratios (ORs) with 95% confidence intervals and associated p-values (significant at p<0.05). Genetic similarity groups and race/ethnicity categories with fewer than 10 individuals were excluded from the logistic regression analyses to avoid unstable estimates. As a sensitivity analysis, Firth’s penalized logistic regression was performed to evaluate the stability of estimates for ancestry groups with small sample size (i.e.: South Asian).

### 2.4 Subgroup analysis

A subgroup analysis was conducted among participants who self-identified as Hispanic. The primary exposure was Indigenous American genetic similarity, evaluated both as a discrete variable (predominant genetic similarity) and as a continuous variable ranging from 0 to 1. Multivariable logistic regression models were used to assess whether Indigenous American genetic similarity was associated with an increased likelihood of receiving a VUS, compared with European genetic similarity. All models were adjusted for the same covariates as the primary analysis, including number of genes tested, testing year, and laboratory.

All analyses were conducted using RStudio (Version 2024.12.1 + 563).

## 3 Results

### 3.1 Participant characteristics

A total of 597 participants were included in the study. Participant characteristics are summarized in **Table 1**. Most participants were female (72%), and the median age was 54 years. Genetic test panels ranged from 6 to 155 genes across multiple commercial laboratories and panel versions; detailed panel composition by laboratory and test name is provided in **Supplementary Table 1**. The most common cancers were breast (33%), colon (25%), and ovarian (8%). Variants of uncertain significance (VUS) were frequent, with 298 participants (50%) receiving one or more VUS, including 58 (9.7%) who received both a VUS and a pathogenic result. Notably, the majority of VUS were concentrated within a core set of standard susceptibility genes (*ATM* (n=31), *BRCA2* (n=26), *APC* (n=26), *PMS2* (n=24)), and *CHEK2* (n=19), which were universally assessed in 99% (591/597) of the cohort.

**Table 1:** Participant characteristics.

Participant characteristics	Number (n=597)
Sex	
Male	167 (28.0%)
Female	430 (72.0%)
Age	
Mean (sd)	54 (13) years
Range	22–89 years
Self-Identified Race/Ethnicity	
Non-Hispanic, White	262 (44%)
Hispanic, White	221 (37%)
Asian	81 (14%)
Non-Hispanic Black	14 (2.4%)
Native Hawaiian or Pacific Islander	2 (0.33%)
More than one	17 (2.9%)
Predominant Genetic Similarity Group	
European	274 (46%)
Indigenous American	160 (27%)
East Asian	79 (13%)
African	22 (3.7%)
Middle Eastern	56 (9.4%)
South Asian	6 (1.0%)
Oceanian	0 (0%)
Genetic test results	
VUS (Total)	298
Positive (Total)	111
Positive (without VUS)	53
Positive (with VUS)	58
Negative	246
Genetic Test Panel Size	
6-20	7 (1.2%)
21 - 35	250 (41.9%)
36 - 50	12 (2.0%)
51 - 65	3 (0.5%)
66 - 79	87 (14.6%)
80 - 94	179 (30.0%)
95 - 109	13 (2.2%)
110 - 124	27 (4.5%)
125 - 139	18 (3.0%)
140 - 155	1 (0.2%)
Cancers	
Breast (including DCIS)	199 (33.3%)
Colon (including colorectal)	149 (25.0%)
Ovarian	50 (8.4%)
Uterus	34 (5.7%)
Prostate	28 (4.7%)
Stomach	15 (2.5%)
Pancreas	14 (2.3%)
Kidney	13 (2.2%)
Skin	13 (2.2%)
Sarcoma	10 (1.7%)
Other*	72 (12.1%)
Testing Lab	
Ambry	184 (30.8%)
Fulgent	46 (7.9%)
LabCorp (Invitae)	144 (24.1%)
Myriad	222 (37.0%)
Univ Washington	1 (0.2%)
Testing Year	
2013	7 (1.2%)
2014	32 (5.4%)
2015	36 (6.0%)
2016	113 (18.9%)
2017	131 (21.9%)
2018	128 (21.4%)
2019	119 (19.9%)
2020	12 (2.0%)
2021	5 (0.8%)
2022	7 (1.2%)
2023	6 (1.0%)
2024	1 (0.2%)

*Including bladder, liver, melanoma, brain, cervix, esophagus, ling, lymphoma, small bowel, thyroid, multiple myeloma and others.

The cohort was diverse in terms of both self-reported race/ethnicity and genetic similarity. Self-identified racial and ethnic composition was as follows: 262 (44%) Non-Hispanic White, 221 (37%) Hispanic White, 81 (14%) Asian, 14 (2%) non-Hispanic Black, 2 (0.3%) Pacific Islander, and 17 (3%) Other/Multiracial. Among Hispanic participants, the majority reported Mexican ancestry on both maternal and paternal sides (n = 143), followed by El Salvadoran (n = 15) and Guatemalan (n = 9), with smaller numbers from other Central and South American countries or mixed/European–Indigenous ancestry (**Supplementary Table 2**). Most Asian participants reported parental ancestry from China (n = 20), the Philippines (n = 18), or Korea (n = 17), with smaller numbers from Japan (n = 6), Vietnam (n = 2), Persia (n = 2), and Sri Lanka (n = 2) (**Supplementary Table 3**). Participants reporting “Unknown/More than one” race/ethnicity (n = 17) reflected broad admixture across Latin American, European, Indigenous American, Asian, Middle Eastern, and African origins (**Supplementary Table 4**).

**Figure 1:**
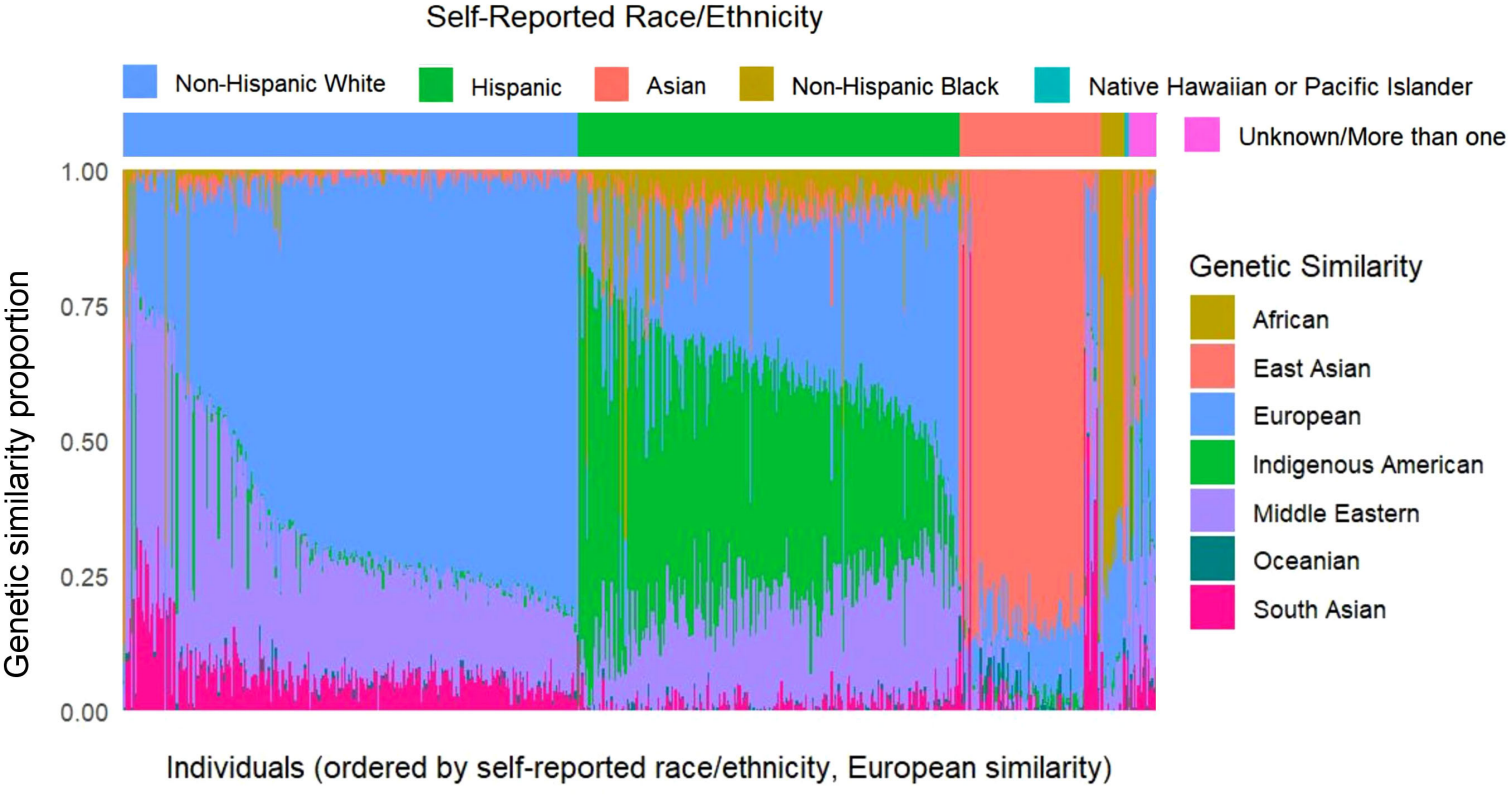
Self-reported race/ethnicity and genetic similarity for study participants. Top: Distribution of participants by self-reported race/ethnicity. Colors correspond to race/ethnicity categories as indicated in the legend. Bottom: Genetic similarity profiles for the same individuals, showing inferred ancestry components. Each vertical bar represents a single individual, with colors corresponding to the proportion of genetic similarity attributed to each ancestral group. This comparison illustrates that self-reported race/ethnicity and genetic ancestry are generally correlated, with notable exceptions for admixed populations such as self-reported Hispanics.

Predominant genetic similarity, defined as the highest similarity estimate in an individual, was distributed as follows: 274 participants (46%) were European, 160 (27%) Indigenous American, 79 (13%) East Asian, 22 (3.7%) African, 56 (9.4%) Middle Eastern, and 6 (1%) South Asian (**Table 1**). Across the cohort (n=597), the mean maximum ancestry proportion was 59.8%, indicating the most individuals had a clearly predominant ancestry component. Only 49 individuals (8.2%) had a difference of ≤5% between their largest and second-largest ancestry proportions and of these, 37 (76%) were self-reported Hispanics, further motivating the secondary analysis examining Indigenous American genetic similarity as a continuous variable. A comparison of self-reported race/ethnicity with genetic similarity proportions for each participant is presented in **Figure 1**.

### 3.2 Association of race/ethnicity and genetic similarity with VUS results

**Figure 2:**
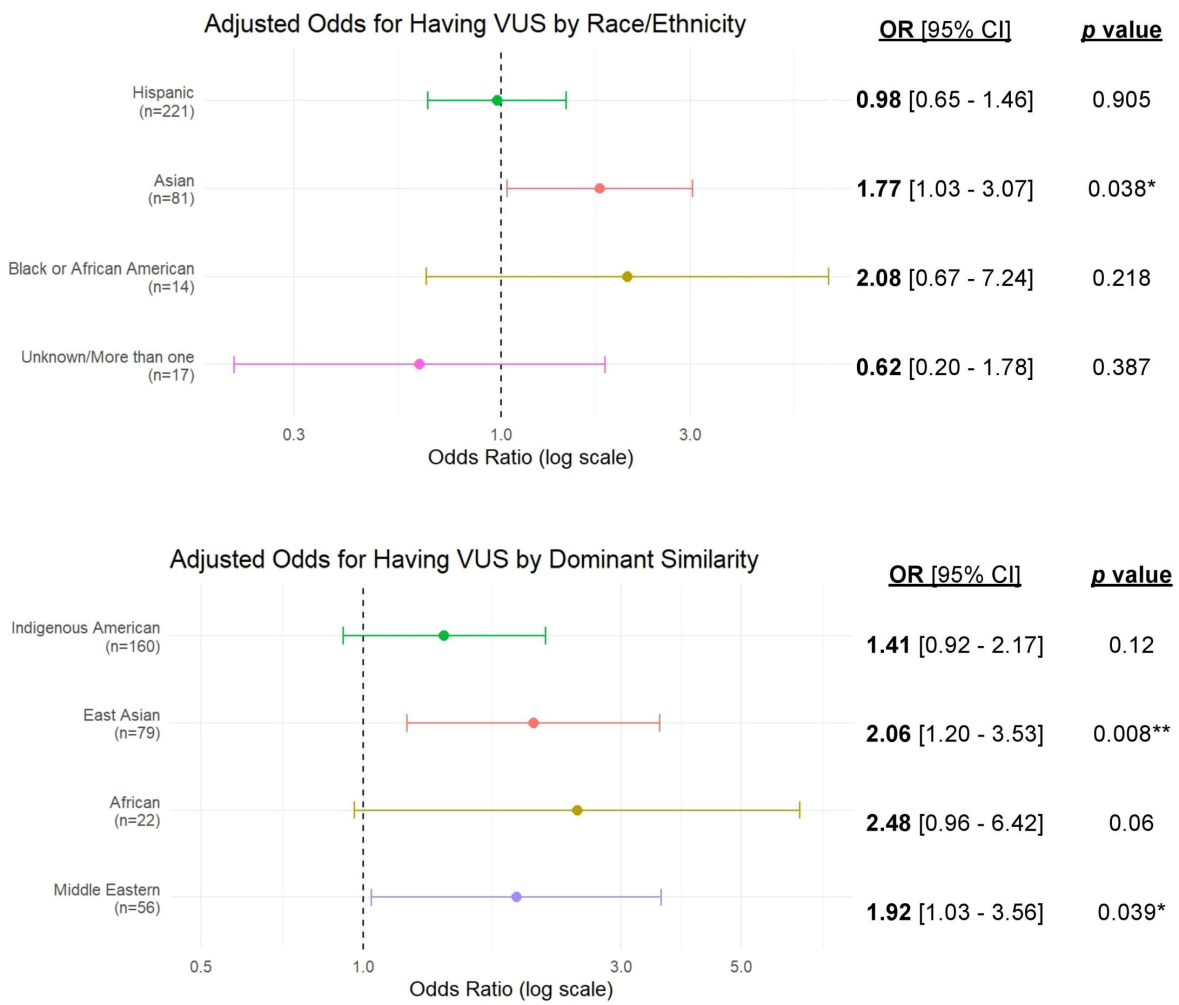
Adjusted odds of having ≥1 variant of uncertain significance (VUS) by self-reported race/ethnicity and genetic similarity. Top: Odds ratios and 95% confidence intervals for the presence of ≥1 VUS by self-reported race/ethnicity (reference group: non-Hispanic Whites, n=262). Bottom: Odds ratios and 95% confidence intervals for the presence of ≥1 VUS by predominant genetic similarity group (reference group: European genetic similarity, n=274). In both cases, the odds ratios were the result of logistic regression, controlling for the number of genes tested, the testing laboratory, and the year of testing.

In univariate analyses, all three covariates (number of genes tested, year of testing, and testing laboratory) were significantly associated with VUS identification (**Table 2**). In multivariable analyses evaluating *self-reported* race/ethnicity, only Asian participants had significantly higher odds of a VUS compared to non-Hispanic White participants (OR = 1.77, 95% CI: 1.03–3.07, p = 0.038) (**Figure 2**). Hispanic participants showed no increased odds (OR = 0.98, 95% CI: 0.65–1.46, p = 0.905). Similarly, those identifying as Unknown/More than one race showed no significant association (OR = 0.62, 95% CI: 0.20–1.78, p = 0.387). Non-Hispanic Black participants had an elevated odds ratio comparable to the Asian cohort, but this did not reach statistical significance (OR = 2.08, 95% CI: 0.67–7.24, p = 0.218).

**Table 2:** Crude (Unadjusted) associations with VUS results.

Variable	Univariate OR (95% CI)	Univariate p-value
Genetic Similarity Group*		
European (reference)	N/A (reference)	N/A (reference)
Indigenous American	1.22 (0.83 - 1.80)	0.32
East Asian	1.88 (1.14 - 3.16)	0.01*
African	2.25 (0.93 - 5.79)	0.08
Middle Eastern	1.84 (1.03 - 3.33)	0.04*
Self-reported Race/Ethnicity**		
Non-Hispanic White (reference)	N/A (reference)	N/A (reference)
Hispanic	0.80 (0.56 - 1.14)	0.22
Asian	1.59 (0.96 – 2.66)	0.07
Non-Hispanic Black	1.77 (0.60 – 5.90)	0.31
More than one	0.54 (0.18 – 1.45)	0.23
Genetic Test Panel Size	1.02 (1.02 - 1.03)	<0.001***
Testing Lab***		
Ambry (reference)	N/A (reference)	N/A (reference)
Fulgent	2.10 (1.06 - 4.37)	0.04*
InVitae	1.26 (0.81 - 1.97)	0.3
Myriad	0.40 (0.27 - 0.60)	<0.001***
Univ Washington*	N/A (too few samples)	N/A (too few samples)
Testing Year	1.25 (1.14 - 1.39)	<0.001***

* South Asian and Oceanian values not reported due to small sample size.

** Native Hawaiian or Pacific Islander values not reported due to small sample size

***Because the University of Washington laboratory represented a sparse factor level (n=1), this category was omitted from regression adjustment, with the observation retained in the analysis.

Analyses using predominant genetic similarity revealed stronger associations (**Figure 2**): compared to European genetic similarity, East Asian (OR = 2.06, 95% CI: 1.20–3.53, p = 0.008) and Middle Eastern (OR = 1.92, 95% CI: 1.03–3.56 p = 0.039) groups had significantly higher odds of a VUS, while African (OR = 2.48, 95% CI:0.96–6.42, p = 0.06) and Indigenous American (OR = 1.41, 95% CI: 0.92–2.17, p = 0.12) groups showed trends toward higher odds. Participants with South Asian (n = 6) or Oceanian (n = 0) genetic similarity were excluded due to low representation. A sensitivity analysis using Firth’s penalized logistic regression (which included the South Asian genetic similarity group, n=6) yielded similar effect estimates and statistical significance patterns, supporting the robustness of the primary findings (**Supplementary Table 5**).

### 3.3 Association of indigenous American ancestry and VUS among Hispanic subgroup

**Figure 3:**
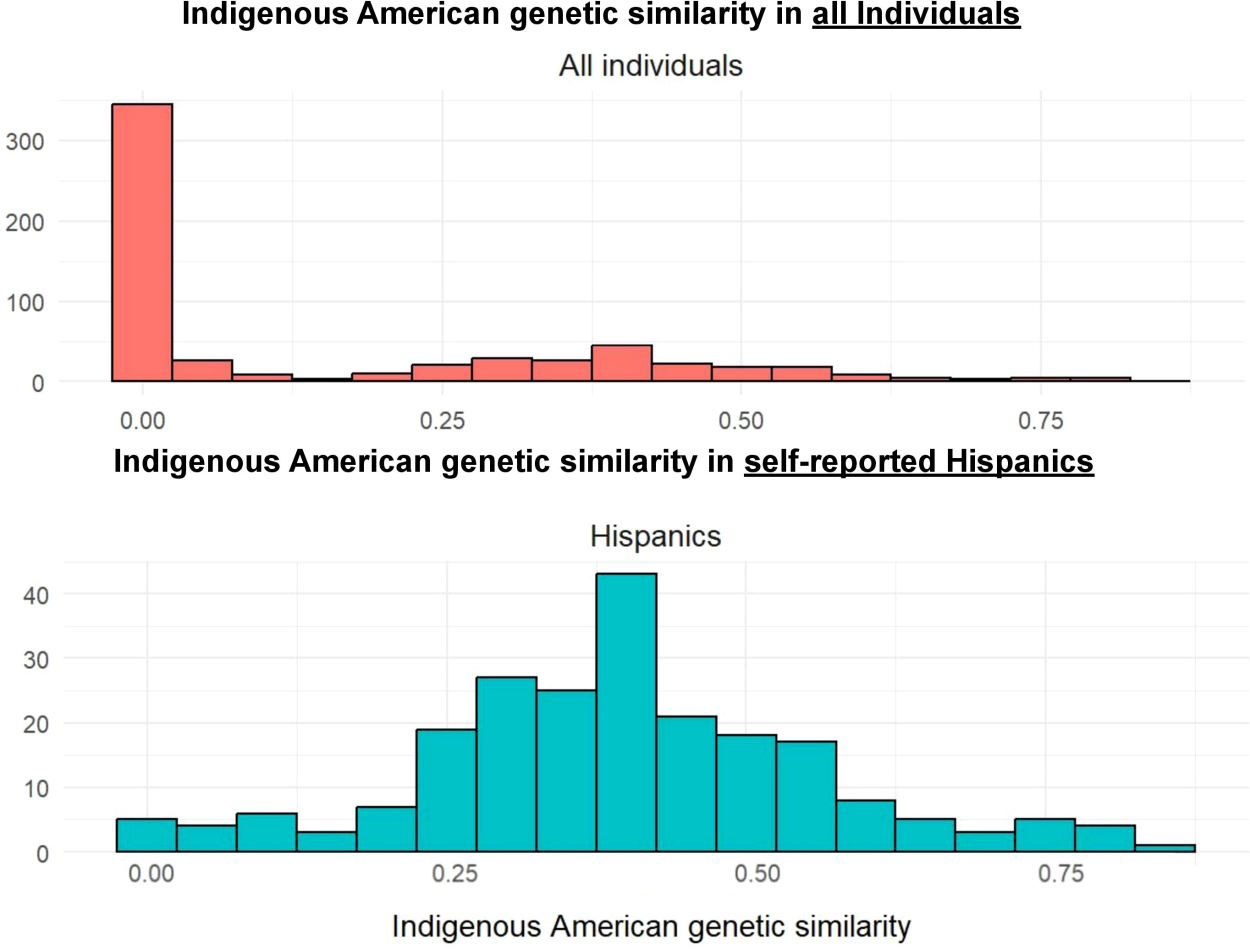
Distribution of Indigenous American genetic similarity in our cohort (n=597) and in the subgroup of self-identified Hispanic participants (n=221). The histogram of Indigenous American ancestry estimated from genetic similarity shows substantial variation within the self-identified Hispanic population, whereas in the overall cohort the distribution is skewed strongly toward lower proportions.

**Figure 4:**
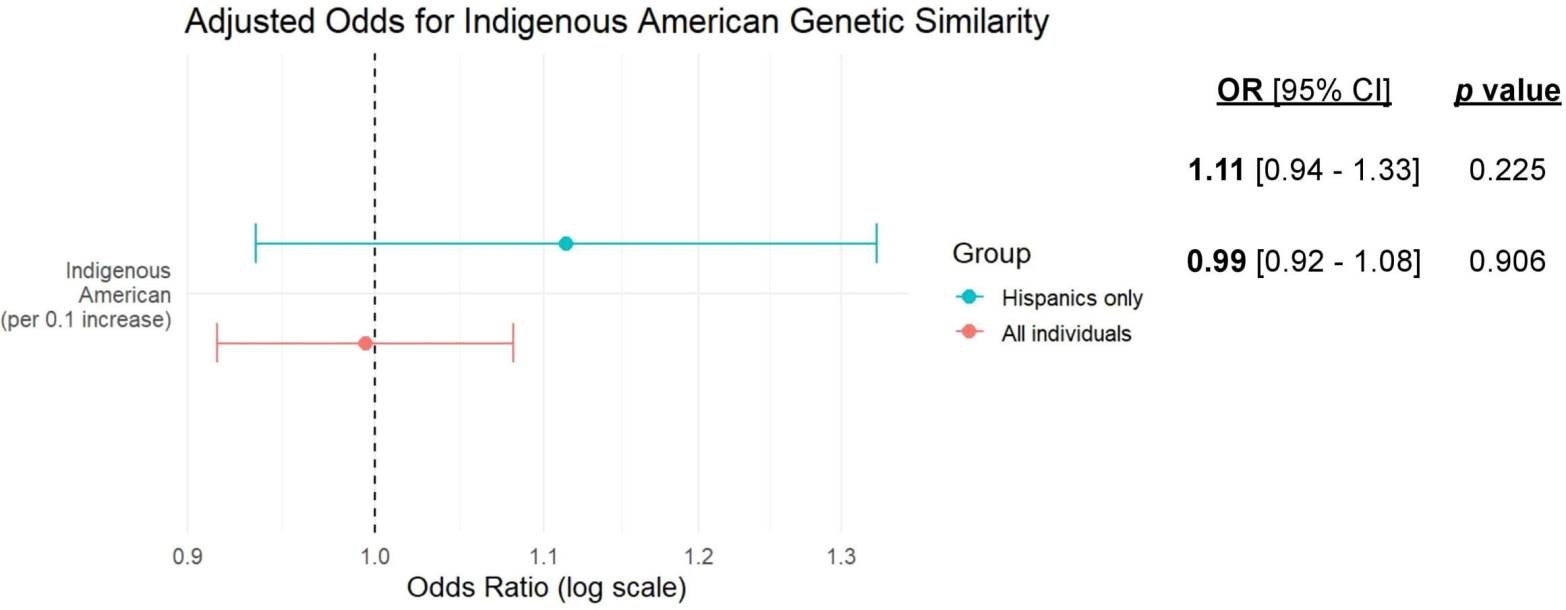
Adjusted odds of VUS by Indigenous American genetic similarity. The forest plot displays the adjusted odds ratios (OR) for identifying a VUS associated with a 10% increase in Indigenous American genetic similarity. Modeled continuously, increasing Indigenous American ancestry was associated with an 11% increase in the odds of a VUS within the self-identified Hispanic subgroup (OR = 1.11, 95% CI: 0.94–1.33), while no such association was observed in the overall cohort (OR = 0.99, 95% CI: 0.92–1.08).

Indigenous American genetic similarity ranged considerably in the whole study population and in the self-identified Hispanic group as demonstrated in the histograms in **Figure 3**. Since self-identified Hispanic individuals had overall higher levels of Indigenous American ancestry, it was possible to explore the effect of Indigenous Ancestry more closely in this population. Those whose predominant genetic similarity was Indigenous American (n=154) had higher odds of receiving a VUS compared to self-identified Hispanics who had predominant European ancestry (n=59) (OR = 1.75, 95% CI = 0.91-3.45, p=0.10). When genetic similarity was measured continuously, every 10% increase in Indigenous American similarity was associated with a 11% increase in odds of a VUS, (OR = 1.11, 95% CI = 0.94-1.33, p=0.225) (**Figure 4**). This effect was not observed in the larger study population (OR = 0.99, 95% CI = 0.92-1.08, p=0.906) (**Figure 4**).

## 4 Discussion

Our primary findings suggest that VUS results are more common in individuals whose genetic ancestry is largely non-European. East Asian and Middle Eastern genetic similarity were associated with nearly double the odds of a VUS compared to European similarity. African genetic similarity showed a similar trend toward higher VUS rates, though the finding did not reach statistical significance, likely due to sample size.

The results also suggest that genetic similarity is more strongly associated with VUS outcomes than self-reported race/ethnicity. In our cohort, the Asian group was the only self-identified category to reach significance, whereas multiple genetic similarity groups showed elevated odds. These results may be explained by the fact that self-identified race/ethnicity and genetic similarity capture different constructs: race/ethnicity is a social identity shaped by historical and sociopolitical contexts, whereas genetic similarity reflects allele frequency patterns shared among populations (24).

While much of the existing literature investigating VUS disparities has relied on clinician-reported or self-reported categories as proxies for ancestry our study utilizes ancestry-informative markers to capture genetic background with greater precision (13, 15, 19, 33–35). This approach is particularly advantageous for studying admixed populations, such as our Hispanic cohort, where categorical labels often fail to capture the full spectrum of genetic diversity. As highlighted in prior work, self-reported race/ethnicity and genetic similarity are often poor proxies for one another (20, 36). For example, some individuals who identify as African American may have predominantly European genetic ancestry (20). Moreover, structural factors in U.S. society, such as limited or monoracial classification options, can obscure admixture and complicate the relationship between identity and ancestry-related insights into genetic risk (37, 38). Using genetic similarity to approximate ancestry provides a nuanced, continuous approach that better reflects the complexity of human genetic variation than discrete categorical labels.

The value of incorporating genetic similarity is further underscored by our Hispanic subgroup analysis. We found that predominant Indigenous American genetic similarity was more strongly associated with VUS results within the Hispanic cohort than in the overall cohort. Furthermore, in an exploratory secondary analysis modeling genetic similarity continuously, we observed a positive trend between Indigenous American ancestry and VUS odds within the Hispanic cohort. While this trend did not reach statistical significance (OR = 1.11, 95% CI = 0.94-1.33, p=0.225), it suggests that for every 10% increase in Indigenous American genetic similarity there is an 11% increase in the odds of a VUS. Notably, this effect was not observed in the overall study population. This highlights the value of exploring genetic heterogeneity within the self-reported Hispanic population. Depending on geographic origin within the US and Latin America, Hispanic-identifying individuals may have varying proportions of European, African, and Indigenous American ancestry and, as a result, varying VUS rates. Similar admixture patterns exist in other populations, such as African Americans and Asians (39, 40).

A major contributor to the disparate VUS rates is the lack of ancestral diversity in the reference and clinical databases used for variant classification. These resources contain an overrepresentation of individuals of European ancestry (12, 17, 41). Consequently, many variants detected in non-European populations are labeled “first observations.” While these may be suspicious for pathogenicity, they cannot be confidently classified without more diverse supporting data (42). One contributor to the lack of ancestral diversity in reference databases is systemic disparities in referral patterns and social determinants of health that restrict genetic testing access for historically marginalized populations (18, 43). While initiatives like gnomAD v4 and *All of Us* are crucial steps toward diversity in genomic reference databases, our results emphasize that databases must account for fine-scale population structure and admixture to reduce misclassifications (41, 44).

Importantly, these considerations extend to computational approaches. While AI-based variant effect predictors may appear agnostic to clinical evidence, they do not circumvent ancestral bias. Most in silico models are trained on the same European-enriched datasets, inheriting historical imbalances in variant reporting (45). As a result, widely used prediction tools continue to exhibit ancestry-related differences in performance and scoring (46). Until training datasets become more representative, computational predictions cannot serve as a fully unbiased alternative and should be interpreted with caution in diverse populations.

The higher rates of VUS in non-Europeans highlight critical disparities in variant classification. Underrepresentation in population databases creates a dual burden for individuals from non-European genetic similarity groups: missed opportunities for risk assessment when pathogenic variants are labeled VUS and potential overtreatment when clinicians take unnecessary measures in response to uncertain findings (47). Provider education on the appropriate evaluation and management of VUS may reduce patient harm, particularly among non-European individuals who are disproportionately more likely to receive VUS results. Genetic counseling for hereditary cancer syndromes typically includes both pre-test and post-test discussions to prepare patients for the possibility of uncertain results and to provide context and support when such results occur (8, 48). Comprehensive VUS counseling should remain standard for patients of any race, ethnicity, or ancestry undergoing cancer genetic testing because any patient can receive VUS results.

### 4.1 Strengths and limitations

The ORIEN-USC dataset offered a robust representation of self-identified Hispanic individuals (37%, n = 221), allowing for a subgroup analysis that many general population studies lack. By modeling genetic similarity as a continuous variable rather than a categorical one, we were able to describe a graded relationship between Indigenous American ancestry proportions and VUS rate in the Hispanic population. Moreover, the unique integration of detailed clinical genetic testing data from CLIA-certified laboratories with genetic ancestry using SNP information provides a powerful resource for exploring ancestry-related patterns in a clinical cancer genetics population, allowing for study of a dilemma in genetic service delivery.

Our study is not without its limitations. Because genetic similarity groups were defined according to the largest genetic similarity estimate, individuals with admixed ancestry were assigned to a single predominant category, which may oversimplify underlying genetic ancestry. Although individuals with near-equivalent ancestry proportions were relatively uncommon, this categorization approach may still obscure important variation within admixed populations. Furthermore, for the self-reported data, in some cases, race/ethnicity was inferred by the clinician based on parental countries of origin documented during pedigree collection. As a result, some individuals in our dataset may have been assigned a race/ethnicity category that did not reflect their self-identification. While this is a bias, it also represents real world scenarios where race/ethnicity by self-report are missing or inferred. Our dataset had comparatively few individuals who identified as African American and South Asian, reflecting the population of the Los Angeles hospitals where they were recruited. This limitation primarily affects the precision of estimates for these subgroups but does not undermine the results relevant to other race, ethnicity, and ancestry groups. Additionally, enrollment into the ORIEN registry itself may introduce selection bias, as it predominantly captures patients who have the resources to access tertiary cancer centers and who are amenable to participating in research initiatives. Future work could expand this analysis to include a larger and more diverse population. While our dataset featured substantial representation of Hispanic participants, our results may not generalize to other Hispanic populations outside of the Los Angeles area with different admixture. Finally, it is worth mentioning that while ancestry-informative markers provide a biologically grounded measure of genetic similarity, the labels assigned to these clusters, such as “Middle Eastern,” are categorical approximations that may overlap with other geographic groups due to historical gene flow and regional admixture. Consequently, these findings should be interpreted as measures of shared genetic variation rather than definitive indicators of geographic origin or self-identity.

## 5 Conclusion

Genetic similarity derived from ancestry-informative markers captured disparities in VUS rates among non-European and admixed populations that were not apparent using self-reported race/ethnicity. Furthermore, we demonstrate a positive relationship between Indigenous American similarity and VUS rates within the Hispanic cohort, which underscores the limitations of using monolithic, categorical labels for admixed populations. To improve accuracy in variant interpretation and mitigate the clinical burden of uncertain findings, it is essential to prioritize the inclusion of diverse, non-European populations in global genomic registries and ensure that all patients receive appropriate post-test counseling and management for VUS results.

## Data Availability

The raw data supporting the conclusions of this article will be made available by the authors, without undue reservation.
