# Supplementary material for: Ancestry-informative markers and variants of uncertain significance on hereditary cancer panels
Source: Front Oncol. 2026 Apr 29;16:1787278. doi: 10.3389/fonc.2026.1787278 (PMC13167527; doi:10.3389/fonc.2026.1787278)
Supplement: Supplementary file 1 [file Table1.docx]

# **Ancestry-Informative Markers and Variants of Uncertain Significance on Hereditary Cancer Panels**

**Supplemental Table 1: Multigene Cancer Panel Characteristics by Testing Laboratory**

| **Name of Lab** | **Name of Test** | **Number of Genes** | **n** |
| --- | --- | --- | --- |
| **Ambry (n=184)** | Ambry BRCAplus v1 | 6 | 1 |
|  | Ambry BRCAplus v3 | 8 | 1 |
|  | Ambry ColoNext v1 | 14 | 1 |
|  | Ambry BreastNext v2 | 16 | 1 |
|  | Ambry RenalNext v2 | 19 | 1 |
|  | Ambry OvaNext v2 | 22 | 1 |
|  | Ambry CancerNext v2 | 24 | 1 |
|  | Ambry CancerNext v3 | 28 | 5 |
|  | Ambry CancerNext v4 | 32 | 10 |
|  | Ambry CancerNext v5 | 34 | 4 |
|  | Ambry Tumor Next with CancerNext | 34 | 5 |
|  | Ambry CancerNext Expanded | 49 | 10 |
|  | Ambry CancerNext Expanded | 67 | 79 |
|  | Ambry CustomNext v1 | 81 | 18 |
|  | Ambry CustomNext v1 (all genes except CFTR) | 80 | 38 |
|  | Ambry CustomNext v2 (without CFTR) | 90 | 3 |
|  | Ambry CustomNext v3 with RNA | 85 | 2 |
|  | Other Ambry panels | 55-79 | 3 |
|  |  |  |  |
| **Myriad (n=222)** | Myriad myRisk v1 | 25 | 92 |
|  | Myriad myRisk v2 | 28 | 115 |
|  | Myriad myRisk v3 | 29 | 4 |
|  | Myriad myRisk v4 | 35 | 10 |
|  | Myriad myRisk v5 | 48 | 1 |
|  |  |  |  |
| **Invitae (Now Labcorp) (n=144)** | Invitae 79 gene Multi-Cancer Panel | 79 | 6 |
|  | Invitae 80 gene Multi-Cancer Panel | 80 | 32 |
|  | Invitae 83 gene Multi-Cancer Panel | 83 | 32 |
|  | Invitae 84 gene Multi-Cancer Panel | 84 | 20 |
|  | Other Invitae panels | 33-155 | 54 |
|  |  |  |  |
| **Fulgent (n=46)** | Fulgent Comprehensive Cancer Panel | 119-127 | 5 |
|  |  |  |  |
| **University of Washington (n=1)** | BROCA | 41 | 1 |

**Supplemental Table 2: Cross-tabulation of Reported Maternal and Paternal Countries of Origin for Participants Reporting Hispanic Race/Ethnicity (n = 221)**

| **Maternal** | **Paternal** | n |
| --- | --- | --- |
| Mexico | Mexico | 143 |
| El Salvador | El Salvador | 15 |
| Guatemala | Guatemala | 9 |
| Mexico/Spain | Mexico | 5 |
| Honduras | Honduras | 4 |
| Nicaragua | Nicaragua | 3 |
| Costa Rica | Costa Rica | 2 |
| Ecuador | Ecuador | 2 |
| El Salvador | Mexico | 2 |
| English/Irish | Mexico | 2 |
| Unspecified | Unspecified | 2 |
| Basque | Basque | 1 |
| Belize | Belize | 1 |
| Belize / Scottish / Mayan | Belize / African / Mayan | 1 |
| British | Lebanese / Hispanic / American Indian | 1 |
| California | Mexico | 1 |
| Costa Rica | Spain | 1 |
| Cuba | Cuba | 1 |
| Cuba | Spain | 1 |
| Egypt | Egypt | 1 |
| El Salvador | Honduras | 1 |
| Germany | Spain | 1 |
| Guatemala | Unspecified | 1 |
| Guatemala, Mexico, Polish | Guatemala | 1 |
| Guatemalan / Spanish | Spanish / Guatemalan / Italian | 1 |
| Honduras | Unspecified | 1 |
| Italy | Argentina | 1 |
| Mexican / Hispanic | Spanish / Italian / English / Native American | 1 |
| Mexican American | Mexican American | 1 |
| Mexican, Indian, Spanish | Mexican, French | 1 |
| Mexico | French/Spanish | 1 |
| Mexico | Germany | 1 |
| Mexico/French | Mexico | 1 |
| Nicaragua | Irish, French, German | 1 |
| Peru | Peru | 1 |
| Peruvian, Bolivian, Italian, Persian | Colombian | 1 |
| Spain/Native American | Mexican | 1 |
| Spanish | African American / Indian / French | 1 |
| Uruguay | Uruguay | 1 |
| USA | USA | 1 |
| W. European/ Cherokee/ Choctaw | Mexican | 1 |
| Welsh/Scandinavian | Mexican/Native American/French | 1 |
| Black/Hispanic/Indian | Black/Hispanic (Nicaragua, Colombia) | 1 |

**Supplemental Table 3: Cross-tabulation of Reported Maternal and Paternal Countries of Origin for Participants Reporting Asian Race/Ethnicity (n = 81)**

| **Maternal** | **Paternal** | n |
| --- | --- | --- |
| China | China | 20 |
| Philippines | Philippines | 18 |
| Korea | Korea | 17 |
| Japan | Japan | 6 |
| Philippines | China | 3 |
| Persia | Persia | 2 |
| Sri Lanka | Sri Lanka | 2 |
| Vietnam | Vietnam | 2 |
| China, Vietnam | Vietnam | 1 |
| Afghanistan | Afghanistan | 1 |
| China | Philippines | 1 |
| India | India | 1 |
| Europe | Japan | 1 |
| Taiwan | Taiwan | 1 |
| Yemen | Yemen | 1 |
| Malaysia | Unspecified | 1 |
| Korea | Uzbek | 1 |
| Persian | Indian | 1 |
| Unspecified | Unspecified | 1 |

**Supplemental Table 4: Cross-tabulation of Reported Maternal and Paternal Countries of Origin for Participants Reporting Unknown/More than One Race/Ethnicity (n = 17)**

| **Maternal ancestry** | **Paternal ancestry** | **Count** |
| --- | --- | --- |
| Portuguese / African | China | 1 |
| Irish, French Canadian, Native American, Scottish, English | French Canadian | 1 |
| Japan | Northern European | 1 |
| American Indian | American Indian / German / Other | 1 |
| England | China | 1 |
| German | China | 1 |
| German | Scottish / Italian / Cherokee | 1 |
| Egyptian (southern) | Egyptian / Moroccan | 1 |
| Eastern European AJ | Moroccan / Israeli | 1 |
| Mixed English | Native American | 1 |
| Native American / Caucasian | Unspecified | 1 |
| Eastern Europe | Unspecified | 1 |
| China | French | 1 |
| Dutch / Caucasian | Spanish | 1 |
| Malaysian | Irish | 1 |
| Mixed English | Native American | 1 |
| Unspecified | Unspecified | 2 |

**Supplemental Table 5: Firth’s Penalized Logistic Regression**

| Ancestry | Odds Ratio | 95% Confidence Interval | p-value |
| --- | --- | --- | --- |
| African | 2.41 | 0.97 - 6.31 | 0.06 |
| East Asian | 2.05 | 1.21 - 3.51 | 0.008 |
| Indigenous American | 1.41 | 0.92 - 2.16 | 0.12 |
| Middle Eastern | 1.9 | 1.04 - 3.52 | 0.04 |
| South Asian | 12.43 | 1.26 - 1686.45 | 0.03 |

Firth’s penalized logistic regression was performed as a sensitivity analysis to address potential bias due to small sample sizes and quasi-complete separation in standard logistic regression models. Effect estimates, 95% confidence intervals, and p-values are shown for each ancestry group. Results were largely consistent with the primary analysis, supporting the robustness of the findings. As expected, penalized regression produced more stable estimates for ancestry groups with small sample sizes, including the South Asian cohort.
